# The impact of oral nicorandil pre-treatment on ST resolution and clinical outcome of patients with acute ST-segment elevation myocardial infarction undergoing primary coronary angioplasty: A randomized placebo controlled trial

**DOI:** 10.34172/jcvtr.2020.16

**Published:** 2020-05-08

**Authors:** Behnaz Akbari, Samad Ghaffari, Naser Aslanabadi, Bahram Sohrabi, Leili Pourafkari, Fariborz Akbarzadeh, Hasan Javadzadegan, Ahmad Separham, Malihe Sehati

**Affiliations:** Cardiovascular Research Center, Tabriz University of Medical Sciences, Tabriz, Iran

**Keywords:** Acute Myocardial Infarction, Oral Nicorandil, ST-Segment Resolution, Primary Angioplasty, Cardioprotection

## Abstract

***Introduction:*** Literature has shown the effects of intravenous/intracoronary nicorandil on increased myocardial salvage in patients with ST-segment elevation myocardial infarction (STEMI) treated with mechanical reperfusion. However, the possible cardioprotective effect of oral nicorandil on the clinical outcome prior to primary coronary angioplasty is not well documented. Our aim was to assess the effect of oral nicorandil on primary percutaneous coronary intervention (PPCI).

***Methods:*** A total of 240 patients with acute STEMI undergoing PPCI were randomly assigned to oral nicorandil (Intervention, n=116) and placebo (Control, n=124) groups. The intervention group received 20 mg oral nicorandil at the emergency department and another 20 mg oral nicorandil in the catheterization laboratory just before the procedure. The control group received matched placebo. Our primary outcome was ST-segment resolution ≥50% one hour after primary angioplasty. Secondary outcome was in-hospital major adverse cardiovascular events (MACE), defined as a composite of death, ventricular arrhythmia, heart failure and stroke.

***Results:*** In the patients of intervention and control groups, the occurrence of ST-segment resolution ≥ 50% were 68.1% and 62.9% respectively, (*P* =0.27). In-hospital MACE occurred less frequently in the intervention group, compared to placebo group (11.2% vs. 22.5%, *P* =0.012).

***Conclusion:*** Although the administration of oral nicorandil before primary coronary angioplasty did not improve ST-segment resolution in patients with acute STEMI, its promoting effects was remarkable on in-hospital clinical outcomes.

***Clinical Registration:*** IRCT20140512017666N1

## Introduction

ST elevation myocardial infarction (STEMI) may be considered as the leading cause of death, worldwide. Mechanical reperfusion by primary percutaneous coronary intervention (PPCI) is reported as the preferred strategy to reduce the infarct size, enhance the left ventricular function, decrease the cardiovascular events and eventually improve the survival of patients. In many patients, however, reperfusion injury develops and myocardial damage increases due to apoptosis and cell death.¹ The reperfusion injury may also lead to an increase in myocardial necrosis and LV dysfunction, which is ultimately accompanied by increasing mortality and hospital complications. Several various medications have been evaluated to prevent and/or treat reperfusion injury, and conflicting results are reported.^2,3^ One of the widely studied drugs is nicorandil. Nicorandil is a nicotinamide ester with features of potassium channel opening and nitrate properties. Its nitrate component causes vasodilatation of the systemic veins and epicardial coronary arteries.^4^ Also, its dual mechanism results in relaxation of smooth muscles of the arteries and veins, and its potassium channel opening feature is responsible for vasodilatation of coronary and peripheral resistance arteries. Therefore, nicorandil increases the blood flow of coronary blood and reduces preload and afterload of the heart.^5^ Previous studies have shown that intravenous or intracoronary nicorandil may have cardioprotective effects and may lead to increased myocardial salvage in patients with STEMI undergoing mechanical reperfusion.^6-10^

However, in a majority of previous studies, nicorandil has been administered either intravenously or intracoronary, and there is paucity of data on possible cardioprotective effect of oral nicorandil before primary coronary angioplasty.^11,12^ Therefore, in the present study, we aimed to assess cardioprotective effect of oral nicorandil during primary angioplasty in patients with STEMI.

## Materials and Methods

This study was approved by the institutional review board in Tabriz University of Medical Science, northwest of Iran (approval code: 58066). All patients completed a written informed consent. This trial has been registered on irct.ir by the number: IRCT20140512017666N1. Between June 2016 and May 2018 we performed a double-blinded randomized clinical trial in the Madani Heart Center, Tabriz, Iran. Patients were eligible if they were 18 years old and higher and admitted with acute STEMI within 12 h of the onset of the symptoms. STEMI was diagnosed based on the following criteria: typical chest pain lasting more than 30 min, ST segment elevation 0.1 mV in at least two contiguous leads and elevated troponin levels.

Exclusion criteria were patients having a permanent pacemaker, left bundle branch block, unsuitable coronary anatomy (candidate for coronary bypass surgery or medical management), hypotension (systolic blood pressure≤ 90 mm Hg) and cardiogenic shock.

**Figure 1:**
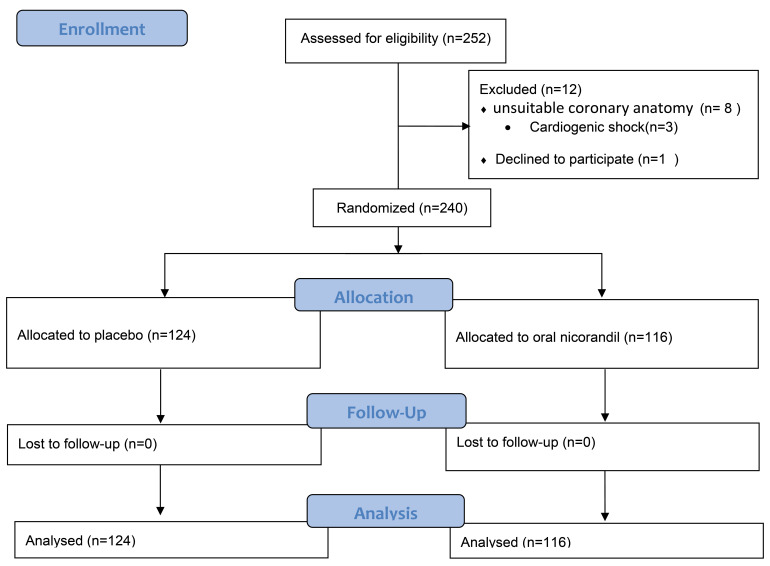
Flow diagram of study (CONSORT).

A total of 252 patients with acute STEMI undergoing PPCI were assessed for eligibility as shown in the CONSORT (Consolidated Standards of Reporting Trials) flow diagram in Figure 1.

Twelve patients were excluded from the study due to unsuitable coronary anatomy (eight patients), cardiogenic shock (three patients) and withdrawing consent (one patient). Finally, 240 patients were randomly assigned to oral nicorandil (intervention, n = 116) and placebo (control, n = 124) groups. The table of random numbers was applied to conduct the randomization. The intervention group received 20 mg oral nicorandil (Ikorel, Sanofi, Paris, France) at the emergency department, and another 20 mg oral nicorandil in the catheterization laboratory just before the procedure. The control group received matched placebo. The physicians in the emergency room and the interventional cardiologist were all blinded to the treatment groups. A 12-lead ECG was recorded on admission and 60 minutes after PCI. ST-segment elevation was recorded 20 msec after the J point. The primary outcome was ST-segment resolution ≥50% one hour after primary angioplasty in the lead with maximal ST-segment elevation in baseline electrocardiogram. ST-segment resolution measurement was done by a physician blinded to the groups. Secondary outcome was in-hospital major adverse cardiovascular events (MACE) which was defined as a composite of death, ventricular arrhythmia, heart failure and stroke. The primary PCI was done via femoral or radial approach. All patients were given oral aspirin (325 mg) and clopidogrel (600 mg) on admission in the emergency room. A bolus of 100 IU/kg of IV heparin was administrated before angioplasty. Thrombectomy device and Eptifibatide use was dependent on operator’s decision. The baseline and post-PCI coronary flows were assessed using the thrombolysis in myocardial infarction (TIMI) flow grading system in the infarct-related artery. Left ventricular ejection fraction (LVEF) applying the Simpson’s method of echocardiography on the second day of admission.

### Statistical Analysis

Based on the findings of study conducted by Ishii et al,^6^ we estimated the incidence of complete ST-segment resolution after PPCI to be 60% in the control and 78% in the intervention groups. A sample size of 100 patients per group was assumed for an alpha level of 0.05% and 80% power using a two-sided test. Assuming a 10% dropout rate, a total patient number of 110 cases per arm was considered. Continuous variables were presented as mean ± standard deviation and qualitative variables as frequency (percentage). Quantitative and qualitative variables were compared applying the student’s *t* test and the chi-square test, respectively. In-hospital clinical outcomes and MACE were analyzed using chi-square test.

SPSS version 16 was used for statistical analysis. The normality of data was evaluated using the Q-Q graph and the Kolmogorov-Smirnov test. *P*  value < 0.05 was considered as statistically significant.

## Results

The baseline characteristics of patients are shown in Table 1. No significant difference was found in age, gender, prevalence of coronary risk factors, history of revascularization and prior medication treatment between the two groups, at baseline. Differences in admission hemodynamic and clinical data were also statistically insignificance at baseline between the intervention and control groups. Procedural characteristics are presented in Table 2. As there is shown in the table, there was no significant difference in location of culprit lesion, multivessel disease, baseline TIMI flow, and thrombus score between the groups.

**Table 1:** Baseline characteristics of patients

	**Nicorandil group (n=116)**	**Placebo group (n=124)**	***P*** **value**
Age (y)	58.9±11.2	57.9±12.7	0.29
Male (n, %)	97 (78.4%)	103 (83.1%)	0.36
Hypertension (n, %)	46 (39.7%)	51 (41.1%)	0.89
Diabetes mellitus (n, %)	25 (21.6%)	23 (18.5%)	0.62
Smoking (n, %)	55 (47.4%)	67 (54%)	0.36
Hyperlipidemia (n, %)	17 (14.7%)	20 (16.1%)	0.85
Previous MI (n, %)	3 (2.6%)	2 (1.6%)	0.67
Previous PCI (n, %)	4 (3.4%)	5 (4%)	0.54
Previous CABG (n, %)	2 (1.7%)	0 (0%)	0.23
Killip class>1 (n, %)	8 (6.7%)	14 (11.2%)	0.26
Door-to-balloon time (min)	60 (45-90)	60 (40-90)	0.60
Systolic blood pressure, mm Hg	136.1±2.37	136.6±2.54	0.11
Heart rate, bpm	97.7±16.2	80±21.8	0.07
Ant MI (n, %)	62 (53.4%)	58 (46.7%)	0.39
LVEF (%)	38.32±6.39	38.16±7.52	0.85
Laboratory data			
Hemoglobin (g/dL)	15.1 (13.3-16.3)	15.3 (13.8-16.5)	0.14
Platelet count (10⁹/ L)	215 (180.5-259)	237 (189-295.2)	0.11
Serum creatinine (mg/dL)	1 (0.97-1.2)	1.1 (1-1.2)	0.11
Glucose (mg/dL)	121 (101-162)	128.5 (103.5-188.5)	0.45
LDL-C (mg/dl)	101 (82-120)	120 (95-141.5)	0.50
Peak Cardiac troponin I (ng/mL)	6.6 (1.6-12.8)	6.1 (1.4-12.3)	0.56
Peak CK-MB (ng/mL)	67.5 (38.7-153.5)	59.5 (33.7-118.5)	0.15
Medications post procedure (n, %)			
Aspirin	116 (100%)	124 (100%)	--
Clopidogrel	116 (100%)	124 (100%)	--
Statins	116 (100%)	124 (100%)	--
β-Blockers	97 (83.6%)	103 (83.06%)	0.17
ACEI/ARB	94 (81.03%)	101 (81.45%)	1.0

Data are given as Mean ± SD, n (%), or median (interquartile range). ACEI, angiotensin-converting enzyme inhibitor; ARB, angiotensin receptor blocker; bpm: beat per minute; CABG, Coronary artery bypass graft surgery; CK-MB, creatinine kinase MB isoenzyme; LDL-C, low-density lipoprotein cholesterol; LVEF, left ventricular ejection fraction; MI, myocardial infarction; PCI, percutaneous coronary intervention.

**Table 2:** Procedural characteristics

	**Nicorandil group (n=116)**	**Placebo group (n=124)**	***P*** **value**
IRA (n, %)			0.3
LAD	62 (53.4%)	58 (46.7%)	
LCX	24 (20.6%)	17 (13.6%)	
RCA	30 (25.8%)	49 (39.5%)	
TIMI flow pre PCI (n, %)			0.85
0	77 (66.3%)	85 (68.5%)	
1	8 (6.8%)	11 (8.8%)	
2	13 (11.2%)	11 (8.8%)	
3	18 (15.1%)	17 (13.7%)	
TIMI flow post-PCI (n, %)			0.38
0	2 (1.7%)	2 (1.6%)	
1	4 (3.4%)	0 (0%)	
2	10 (8.6%)	10 (8.06%)	
3	100 (86.2%)	112 (90.3%)	
Number of diseased vessel, n (%)			0.60
SVD	49 (42.2%)	55 (44.3%)	
Multivessel disease	67 (57.8%)	69 (55.7%)	
TMPG pre-PCI (n, %)			0.79
0	69 (59.5%)	76 (61.3%)	
1	28 (24.1%)	32 (25.8%)	
2	13 (11.2%)	11 (8.8%)	
3	6 (5.1%)	5 (4.03%)	
TMPG post-PCI (n, %)			0.71
0	5 (4.3%)	4 (3.2%)	
1	4 (3.4%)	5 (4.03%)	
2	8 (6.8%)	7 (5.6%)	
3	99 (85.3%)	108 (87.09%)	
Drug-eluting stent (n, %)	66 (62.9%)	71 (63.4%)	0.65
Intracoronary Eptifibatide (n, %)	14 (12.06%)	18 (14.5%)	0.70
Thrombectomy (n, %)	2 (1.7%)	3 (2.4%)	1.0
Thrombus scores, n (%)			0.76
0	14 (12.1%)	8 (6.5%)	
1	10 (8.6%)	10 (8.1%)	
2	6 (5.2%)	8 (6.5%)	
3	5 (4.3%)	7 (5.7%)	
4	9 (7.8%)	11 (8.9%)	
5	72 (62.1%)	79 (64.2%)	
Stent diameter (mm)	3.07±0.05	3.06±0.03	0.059
Stent length (mm)	20.01±0.1	19.94±0.95	0.41

Data are given as Mean ± SD, n (%). IRA: Infarct related artery; LAD: left anterior descending artery; LCX - left circumflex artery; RCA: right coronary artery; PCI: percutaneous coronary intervention; TIMI: Thrombolysis in Myocardial Infarction; SVD: Single vessel disease; TMPG: TIMI myocardial perfusion grade.

Moreover, intracoronary eptifibatide and thrombectomy catheter use was similar between the intervention and control groups. Eleven patients in the intervention group and 12 patients in the control group received only balloon angioplasty.

**Figure 2:**
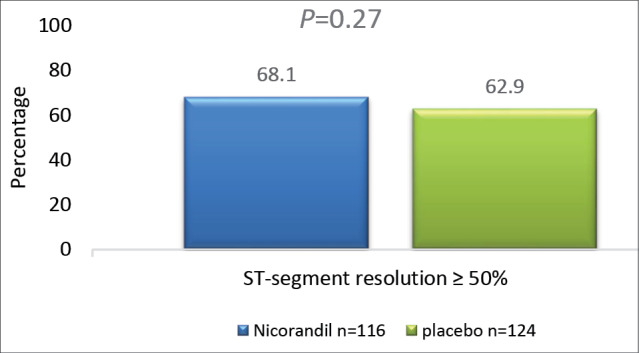
Complete ST-segment resolution in the study groups.

Regarding the primary outcome, complete ST-segment resolution (equal or more than 50%) occurred in 68.1% of patients in the intervention and 62.9% of patients in the control groups (*P* value = 0.27) (Figure 2) .As the secondary outcome, in-hospital MACE occurred less frequently in the intervention group compared to the control group (11.2% vs 22.5% , *P* value = 0.012) (Table 3).

**Table 3:** In-hospital MACE (n, %)

	**Nicorandil group** **(n=116)**	**Placebo group (n=124)**	***P*** **value**
Heart failure	8 (6.8%)	14 (11.3%)	0.26
VT/VF	4 (3.4%)	11 (8.9%)	0.11
Stroke	1 (0.9%)	1 (0.8%)	1.0
Death	0	2 (1.6%)	0.49
Total	13 (11.2%)	28 (22.5%)	0.012

MACE: Major adverse cardiovascular event; VT: Ventricular tachycardia; VF: Ventricular fibrillation.

## Discussion

Our aim in the present study was to assess the effect of oral nicorandil on PPCI among the patients with ST-segment elevation myocardial infarction (STEMI) treated with mechanical reperfusion.

The main findings of our study were as follow: 1) The two-stage oral administration of nicorandil (two doses of 20 mg) in emergency department and immediately before coronary angioplasty did not have any improving effect on of ST-segment resolution 2) the nicorandil intervention before primary angioplasty, however improved in-hospital outcomes of patients with ST-segment elevation treated with primary coronary angioplasty.

In the present study, ST-segment resolution ≥ 50%, as a major determinant of reperfusion success, was not improved by oral nicorandil prior to PPCI. This finding is in contrary to those found in the most of previous studies where the drug was used through either intravenous or intracoronary route.^6-9,13,14^ Also, in the present study, oral nicorandil did not have any effect on infarct size reduction and left ventricular function as evident by similar peak cardiac troponin and left ventricular ejection fraction (Table 1) . These findings are consistent with those reported by Kitakaze et al, who showed the intravenous nicorandil with no effect on either infarct size or left ventricular function. Notably, in their study, the patients who were given chronic oral nicorandil had a better level of left ventricular function at long-term follow up, an outcome that was not evaluated in the present study.^15^ Although our findings had similarities with those reported in the studies conducted on the patients with stable and unstable angina,^16,17^ we failed to show any beneficial effect of oral nicorandil on coronary blood flow after angioplasty, as evident in similar TIMI flow 3 rate (Table 2). This finding of our study was in contrast to the most of reports on the reduction of no-reflow phenomenon with either intravenous or intracoronary nicorandil administration.^8,10,18-20^

The following mechanism may be responsible for this lack of ischemia/reperfusion injury reduction in oral nicorandil as applied in the present study: longer absorption time of oral nicorandil compared to intravenous or intracoronary forms, which may lead to delayed delivery of the medication to myocardium and thus impeding adequate serum levels for cardioprotection during primary angioplasty. Larger oral doses and increasing the times of administration may be promote the effects of the medicine, but could potentially deteriorate hemodynamic status of the patients, due to the hypotensive effect of nicorandil.

On the other hand, in-hospital outcomes (lower levels of ventricular arrhythmia and heart failure) of the patients who received oral nicorandil were better than the placebo. As a potassium channel opener, nicorandil shortens action potential duration and reduces Calcium overload in ischemic myocardium which leads to suppression of ventricular arrhythmia.^21-23^ Similar studies have reported the positive effects of oral nicorandil in the setting of acute reperfused STEMI.

Although we found no reduction in the infarct size, our finding on the successful effect of oral nicorandil on preventing heart failure development was noteworthy. The precise mechanism is not clear, as it cannot be explained by the improvement in coronary blood flow and reduction in no-reflow phenomenon. However, apart from the preconditioning and antiapoptotic effect of nicorandil via mitochondrial ATP-sensitive potassium channels, some other possible mechanisms about cardioprotection of nicorandil may be concluded.^24^ In a rat model of myocardial ischemia reperfusion injury, nicorandil exerted its cardioprotective effect via the inhibition of endoplasmic reticulum (ER) stress induced apoptosis and cell death through the PI3K/Akt (phosphatidylinositol 3-kinase (PI3K) pathway.^25^ Another possible mechanism may be related to anti-fibrotic effect of nicorandil via the inhibition of 70-kDa S6 (p70S6) kinase pathway.^26^ Nicorandil also shifts the macrophage phenotype and leads to less myofibroblast differentiation through the inhibition of RhoA/RhoA-kinase-dependent pathway.^27^ Myofibroblast is suggested to be involved in the progression of left ventricular remodeling and arrhythmogenic activity.^28,29^

This suggestion, however, is only a hypothesis-generating interpretation and needs further larger human studies for confirmation.

Our study had several limitations: First, small sample size precluded the definite conclusion regarding the role of oral nicorandil before primary coronary angioplasty. Second, this was a single center study with no different ethnical population. Third, we did not perform any precise infarct size measures, like cardiac magnetic resonance imaging. Fourth, long-term follow up data was not available.

As previous studies have relatively approved the efficacy of chronic oral nicorandil administration in STEMI patients, we did not continue oral nicorandil administration during hospital stay and after discharge.^12,21,30^ So, further large scale multicenter randomized control trials with long-term follow ups are recommended.

## Conclusion

Administration of oral nicorandil before primary coronary angioplasty did not improve ST-segment resolution, left ventricular function, enzymatic infarct size and coronary blood flow in patients with acute STEMI. However, irrespective to the effects of nicorandil on mechanical reperfusion success, the intervention showed promise in the improvement of in-hospital clinical outcomes.

## Competing interests

The authors declare that they do not have any conflicts of interest about this work.

## References

[1] Kloner RA (1993). Does reperfusion injury exist in humans?. J Am Coll Cardiol.

[2] Tonet E, Bernucci D, Morciano G, Campo G (2018). Pharmacological protection of reperfusion injury in ST-segment elevation myocardial infarction. Gone with the wind? Postepy Kardiol Interwencyjnej.

[3] Campo G, Pavasini R, Morciano G, Lincoff AM, Gibson CM, Kitakaze M (2017). Clinical benefit of drugs targeting mitochondrial function as an adjunct to reperfusion in ST-segment elevation myocardial infarction: A meta-analysis of randomized clinical trials. Int J Cardiol.

[4] Schmid J-P, Schroeder V (2005). Nicorandil–review of pharmacological properties and clinical applications. Heart Drug.

[5] Frydman A (1992). Pharmacokinetic profile of nicorandil in humans: an overview. J Cardiovasc Pharmacol.

[6] Ishii H, Ichimiya S, Kanashiro M, Amano T, Imai K, Murohara T (2005). Impact of a single intravenous administration of nicorandil before reperfusion in patients with ST-segment-elevation myocardial infarction. Circulation.

[7] Feng C, Han B, Liu Y, Wang L, Niu D, Lou M (2018). Effect of nicorandil administration on myocardial microcirculation during primary percutaneous coronary intervention in patients with acute myocardial infarction. Postepy Kardiol Interwencyjnej.

[8] Qi Q, Niu J, Chen T, Yin H, Wang T, Jiang Z (2018). Intracoronary nicorandil and the prevention of the no-reflow phenomenon during primary percutaneous coronary intervention in patients with acute ST-segment elevation myocardial infarction. Med Sci Monit.

[9] Feng C, Liu Y, Wang L, Niu D, Han B (2019). Effects of early intracoronary administration of nicorandil during percutaneous coronary intervention in patients with acute myocardial infarction. Heart Lung Circ.

[10] Wu M, Huang Z, Xie H, Zhou Z (2013). Nicorandil in patients with acute myocardial infarction undergoing primary percutaneous coronary intervention: a systematic review and meta-analysis. PLoS One.

[11] Yang J, Zhang J, Cui W, Liu F, Xie R, Yang X (2015). Cardioprotective effects of single oral dose of nicorandil before selective percutaneous coronary intervention. Anatol J Cardiol.

[12] Sakata Y, Nakatani D, Shimizu M, Suna S, Usami M, Matsumoto S (2012). Oral treatment with nicorandil at discharge is associated with reduced mortality after acute myocardial infarction. J Cardiol.

[13] Yamada K, Isobe S, Ishii H, Yokouchi K, Iwata H, Sawada K (2016). Impacts of nicorandil on infarct myocardium in comparison with nitrate: assessed by cardiac magnetic resonance imaging. Heart Vessels.

[14] Suematsu Y, Murasato Y, Miura S, Horiuchi M, Yamamoto T, Takata K (2013). Safety and feasibility of high-dose administration of nicorandil before reperfusion therapy in acute myocardial infarction. Cardiovasc Interv Ther.

[15] Kitakaze M, Asakura M, Kim J, Shintani Y, Asanuma H, Hamasaki T (2007). Human atrial natriuretic peptide and nicorandil as adjuncts to reperfusion treatment for acute myocardial infarction (J-WIND): two randomised trials. Lancet.

[16] Hwang J, Lee HC, Kim BW, Yang MJ, Park JS, Park JH (2013). Effect on periprocedural myocardial infarction of intra-coronary nicorandil prior to percutaneous coronary intervention in stable and unstable angina. J Cardiol.

[17] Ejiri K, Miyoshi T, Kohno K, Nakahama M, Doi M, Munemasa M (2018). Protective effect of remote ischemic preconditioning on myocardial damage after percutaneous coronary intervention in stable angina patients with complex coronary lesions -subanalysis of a randomized controlled trial. Circ J.

[18] Pang Z, Zhao W, Yao Z (2017). Cardioprotective Effects of Nicorandil on Coronary Heart Disease Patients Undergoing Elective Percutaneous Coronary Intervention. Med Sci Monit.

[19] Lee HC, An SG, Choi JH, Lee TK, Kim J, Kim JH (2008). Effect of intra-coronary nicorandil administration prior to reperfusion in acute ST segment elevation myocardial infarction. Circ J.

[20] Ono H, Osanai T, Ishizaka H, Hanada H, Kamada T, Onodera H (2004). Nicorandil improves cardiac function and clinical outcome in patients with acute myocardial infarction undergoing primary percutaneous coronary intervention: role of inhibitory effect on reactive oxygen species formation. Am Heart J.

[21] Ito H, Taniyama Y, Iwakura K, Nishikawa N, Masuyama T, Kuzuya T (1999). Intravenous nicorandil can preserve microvascular integrity and myocardial viability in patients with reperfused anterior wall myocardial infarction. J Am Coll Cardiol.

[22] Kobayashi Y, Miyata A, Tanno K, Kikushima S, Baba T, Katagiri T (1998). Effects of nicorandil, a potassium channel opener, on idiopathic ventricular tachycardia. J Am Coll Cardiol.

[23] Wang YP, Zhang Y, Sun YR, Sun ZG, Zuo ZK, Feng ZR (2017). Effect of nicorandil on ventricular arrhythmia in patients with acute ST-segment elevation myocardial infarction underwent emergent percutaneous coronary intervention treatment. Zhonghua Xin Xue Guan Bing Za Zhi.

[24] Akao M1, Teshima Y, Marbán E (2002). Antiapoptotic effect of nicorandil mediated by mitochondrial atp-sensitive potassium channels in cultured cardiac myocytes. J Am Coll Cardiol.

[25] Wu H, Ye M, Yang J, Ding J, Yang J, Dong W (2015). Nicorandil Protects the Heart from Ischemia/Reperfusion Injury by Attenuating Endoplasmic Reticulum Response-induced Apoptosis Through PI3K/Akt Signaling Pathway. Cell Physiol Biochem.

[26] Lee TM, Lin MS, Chang NC (2008). Effect of ATP-sensitive potassium channel agonists on ventricular remodeling in healed rat infarcts. J Am Coll Cardiol.

[27] Lee TM, Lin SZ, Chang NC (2018). Nicorandil regulates the macrophage skewing and ameliorates myofibroblasts by inhibition of RhoA/Rho-kinase signalling in infarcted rats. J Cell Mol Med.

[28] Davis J, Molkentin JD (2014). Myofibroblasts: trust your heart and let fate decide. J Mol Cell Cardiol.

[29] Askar SF, Ramkisoensing AA, Schalij MJ, Bingen BO, Swildens J, van der Laarse A (2011). Antiproliferative treatment of myofibroblasts prevents arrhythmias in vitro by limiting myofibroblast-induced depolarization. Cardiovasc Res.

[30] Wang S, Duan Y, Feng X, Liu L, Shi Z, Wang B (2019). Sustained nicorandil administration reduces the infarct size in ST-segment elevation myocardial infarction patients with primary percutaneous coronary intervention. Anatol J Cardiol.

